# The impact of scoliosis surgery on pulmonary function in spinal muscular atrophy: a systematic review

**DOI:** 10.1007/s43390-021-00302-w

**Published:** 2021-03-08

**Authors:** Abduljabber Alhammoud, Yahya Othman, Ron El-Hawary, William G. Mackenzie, Jason J. Howard

**Affiliations:** 1 Hamad Medical Corporation, Doha, Qatar; 2 Kaiser Permanente Oakland Medical Center, Oakland, CA USA; 3 Weill Cornell Medical College, Education City, Doha, Qatar; 4 IWK Health Center, Division of Orthopedic Surgery, Dalhousie University, 5850/5980 University Ave, Halifax, NS B3K 6R8 Canada; 5 Department of Orthopedic Surgery, Nemours/Alfred I. duPont Hospital for Children, 1600 Rockland Road, Wilmington, DE 19803 USA

**Keywords:** Spinal muscular atrophy, Scoliosis, Pulmonary function, Systematic review, Post-operative complications

## Abstract

Scoliosis often occurs coincident with pulmonary function deterioration in spinal muscular atrophy but a causal relationship has not yet been reliably established. A systematic literature review was performed, with pulmonary function testing being the primary outcome pre- and post-scoliosis surgery. Levels of evidence were determined and GRADE recommendations made. Ninety studies were identified with only 14 meeting inclusion criteria. Four studies were level III and the rest were level IV evidence. The average age at surgical intervention was 11.8 years (follow-up 6.1 years). Post-operative pulmonary function progressively declined for the majority of studies. Otherwise, pulmonary function: improved (two studies), were unchanged (two studies), had a decreased rate of decline (three studies), declined initially then returned to baseline (two studies). Respiratory and spine-based complications were common. Given the available evidence, the following GRADE C recommendations were made: (1) surgery is most often associated with decreases in pulmonary function; (2) the impact of surgery on pulmonary function is variable, but does not improve over pre-operative baseline; (3) surgery may result in a decreased rate of decline in pulmonary function post-operatively. Given this lack of evidence-based support, the risk–benefit balance should be taken into consideration when contemplating scoliosis surgery.

## Introduction

Spinal muscular atrophy (SMA) is an autosomal recessive neuromuscular disorder caused by mutations or deletions in the SMN1 gene. The SMN1 gene is responsible for the production of the survival motor neuron (SMN) protein which, when defective, is associated with the degeneration of cell bodies within the alpha motor neurons of the anterior horn of the spinal cord. This results in a symmetrical lower motor neuron syndrome, typified by hypotonia, diminished reflexes, fasciculations, progressive muscle atrophy, and weakness [29]. Proximal muscle weakness is more prevalent in SMA—with the lower extremities more involved than the upper extremities—along with progressive weakness of the muscles responsible for respiration [12, 13]. Progression of scoliosis and chest wall deformity is often coincident with deterioration in pulmonary function, but a causal relationship has not yet been reliably established. Accordingly, the impact of scoliosis surgery on pulmonary function remains controversial.

Given the impact of concomitant decreases in respiratory dysfunction on quality of life and life expectancy, many surgeons are treating scoliosis in SMA aggressively and at an early stage; with the hope that this strategy will significantly alter natural history [12, 30, 34]. Improvements spinal/chest wall implant systems, peri-operative critical care management, and the promising results of medical therapies such as intrathecal Nusinersen, have helped encourage this enthusiasm [11, 22].

Despite these advances, scoliosis surgery in SMA carries a high risk of complications in this medically frail population [2]. As such, the indications for surgery need to be clarified, particularly with respect to its impact on pulmonary function. Hence, the goal of this review was to determine the impact of scoliosis correction on pulmonary function in SMA, to gauge whether an aggressive surgical approach is warranted given the risks associated.

## Materials and methods

Following the Preferred Reporting Items for Systematic Reviews and Meta-Analyses (PRISMA) guidelines [25], a systematic literature review was performed, collecting studies published as of February 2020. PubMed/MEDLINE and Google Scholar databases were searched for relevant articles. The following search terms “(scoliosis) AND (spinal muscle atrophy or SMA or spinal atrophy) AND (respiratory or PFT or pulmonary function test)” were utilized. Only studies reporting pre- and post-operative pulmonary function test (PFT) results for paediatric patients with SMA who have undergone scoliosis surgery were included. Studies involving other neuromuscular diagnoses or did not report results of scoliosis surgery and PFTs, were excluded. A manual search of the reference lists of the selected articles was performed to identify additional articles for inclusion. Searches were limited to include only English-language studies.

The primary outcome was pulmonary function. Secondary outcomes included percent curve correction, surgical approach, type of surgery, age at surgery, and complication rates. Additionally, data was collected for: study characteristics (study name, publication date, country of origin and level of evidence [32]) and patient demographics (age at surgical intervention, sex, follow-up period, SMA Type).

All citations identified in the search had their titles and abstracts assessed by two reviewers working independently (AA and JJH). Studies where abstracts are deemed potentially relevant were assessed in full-text format to assess for inclusion for analysis based on pre-defined criteria. Disagreements were resolved by discussion.

When assessing retrieved studies designated for inclusion, the *Journal of Bone and Joint Surgery* level-of-evidence ratings were utilized [15]. Evidence-based statements, each qualified with a *Grade of recommendation*, were subsequently developed according to Wright and colleagues [33]. These Grades were defined as follows:

- GRADE A—good evidence based on level I studies with consistent findings.
- GRADE B—fair evidence based on consistent level II or Level III studies.
- GRADE C—poor or conflicting evidence based on level IV/V studies.
- GRADE I—insufficient evidence to make a treatment recommendation.

## Results

**Fig. 1:**
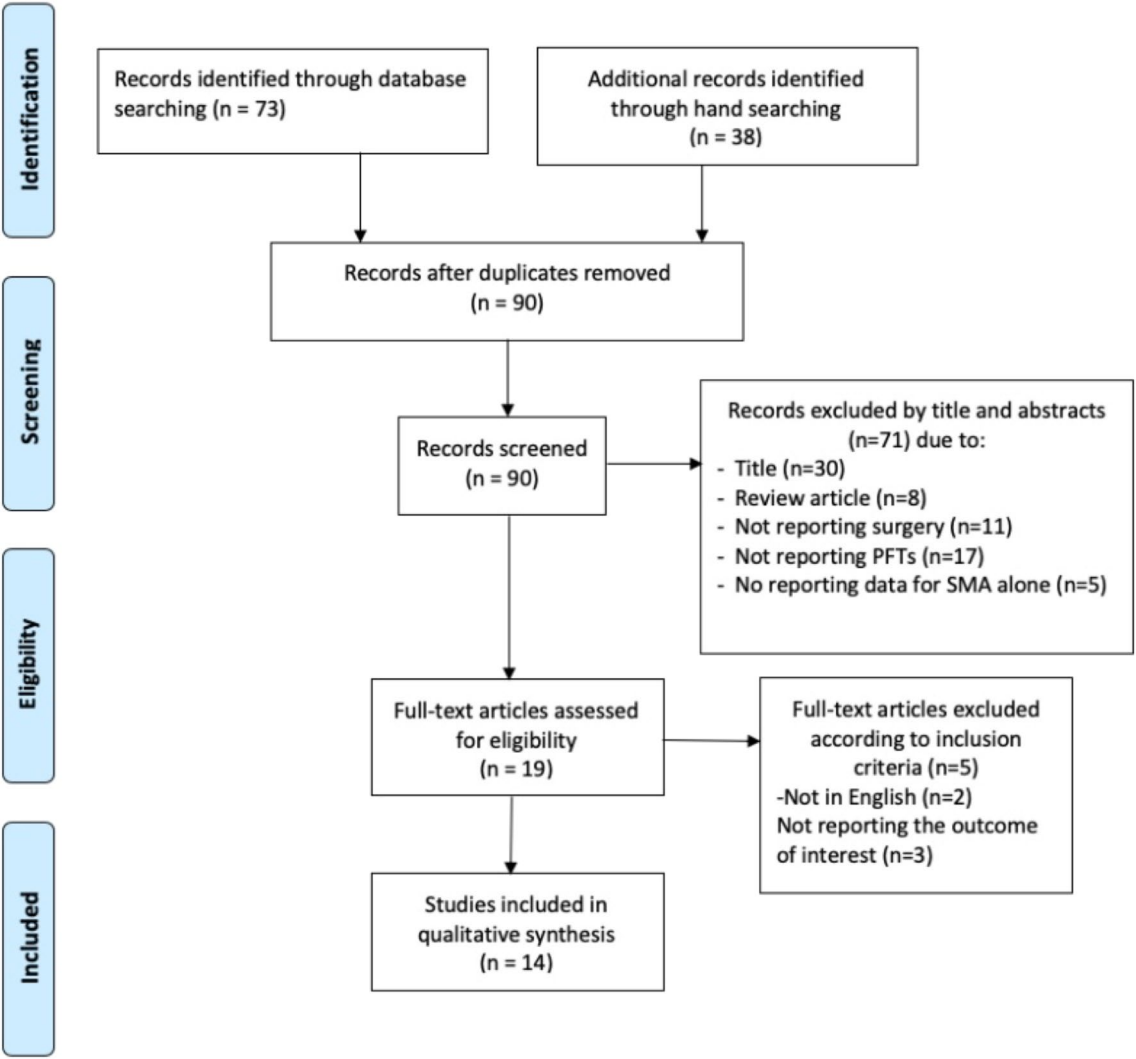
PRISMA flow diagram [7]

The initial database(s) search revealed 90 studies after the removal of duplicates. Seventy-one studies were excluded after reviewing the title/abstract and applying the inclusion criteria. A flow diagram detailing our search strategy using the PRISMA framework, and determination of included studies, is provided in Fig. 1. The level of evidence for 10 of 14 included studies identified was IV, with the rest being level III.

The included studies involved a total of 242 subjects with SMA, with an average of 17 patients per study. The average age at surgical intervention was 11.8 years. The mean follow-up period was 6.1 years. Only 8 of 14 studies reported the types of SMA and of these 12% were Type I, 71% were Type II, and 17% were Type III.

The majority of surgical interventions were performed through a posterior approach (96%) while only 4% of patients had anterior procedures. Operations performed posteriorly included Luque sublaminar wiring and Galveston pelvic fixation, Harrington instrumentation, segmental pedicle screw instrumentation, and growing rods (including magnetically-controlled, traditional growing rods, and vertical expandable titanium rib). The only anterior approach technique reported was the Dwyer anterior instrumentation and fusion. The mean curve correction percentage was 51% with a range of 25–65.0% reported. A full summary of demographic data from the included studies is reported in Table 1.

**Table 1:** Demographic data of included studies

Study name	Level of evidence	Country of origin	# Of patients per study	Average age at surgery (years)	Follow up (years)	SMA type	Type of surgery	Curve correction
Aprian 1982 [1]	IV	USA	22	12.2 y	6.8	I: 7 II: 12 III: 3	Posterior: 16 SSI/H Anterior: 6 D	44%
Piasecki 1986 [26]	IV	USA	19	15.6	NR	NR	Posterior: 19 SSI/H Anterior: 0	25%
Brown 1989 [3]	III	USA	40	11.3	4.3	NR	Posterior: 40 LG/H Anterior: 0	42%
Merlini 1989 [23]	IV	Italy	7	15.3	3	I: 0 II: 4 III: 3	Posterior: 7 LG/H Anterior: 1 D	45%
Granata 1993 [14]	IV	Italy	18	16.0	5	NR	Posterior: 15 LG/H Anterior: 3 D	50%
Robinson 1995 [28]	IV	UK	16	12.1	NR	NR	Posterior: 16 SSI/H Anterior: 0	40%
Chng 2003 [4]	IV	Singapore	8	9.7	3.8	I: 0 II: 4 III: 4	Posterior: 8 G/L Anterior: 0	65%
Chong 2010 [5]	III	Korea	11	NR	1.9	NR	Posterior: 11 SSI Anterior: 0	48%
Fujak 2012 [12]	III	Germany	41	9.9	8.6	I: 0 II: 37 III: 4	Posterior: 41 TR/SSI Anterior: 0	61%
Chou 2017 [6]	IV	Taiwan	10	12.9	12.3	I: 0 II: 10 III: 0	Posterior: 9 SSI/LG Anterior: 1 D	61%
Chua 2016 [7]	III	Singapore	11	9.1	11.6	I: 0 II: 8 III: 3	Posterior: 11 G/L Anterior: 0	44%
Lenhart 2017 [19]	IV	USA	16	5.8	4.7	I: 5 II: 11 III: 0	Posterior: 16 TR Anterior: 0	62%
Holt 2017 [15]	IV	USA	16	9.8	10.1	I: 3 II: 8 III: 5	Posterior: 16 SSI/LG/H Anterior: 0	65%
Farber 2020 [10]	IV	USA	6	13.9	1.1	NR	Posterior: 6 INS Anterior: 0	57%
Total			Mean: 17 per study Total: 242	11.8	6.1	I: 12% II: 71% III:17%	Posterior: 96% Anterior: 4%	51%

*NR* not reported, *D* Dwyer, *LG* Luque–Galveston, *H* Harrington, *SSI* segmental screw instrumentation, *TR* telescopic rod, *INS* instrumentation not specified

All included studies reported pulmonary function testing results both pre-operatively and post-operatively. Forced vital capacity (FVC) and vital capacity (VC) were most commonly reported PFT measures by either absolute or percent predicted values or both. Summary statements regarding pulmonary function outcomes for each of the included studies is presented in Table 2. The majority of included studies reported a progressive decline in PFTs post-operatively without reference to the change in the rate of decline from pre-operative status. [1, 3, 5, 10, 26] Three studies reported a decreased rate of decline in PFTs post-operatively compared to the pre-operative rate [4, 7, 15]. Two studies demonstrated an initial decrease in PFTs immediately post-operatively, followed by a return to pre-operative values within 12 months [12, 14]. Two studies reported no difference between pre-operative and post-operative PFTs following a posterior-only approach. [6, 23] One study correlated PFT values according to the surgical approach, reporting a significant decrease in VC (40%) following an anterior approach, but no change in VC with the posterior approach alone [6].

**Table 2:** Pulmonary function tests (PFT) results statements summarizing the results from included studies

Study name	Type of PFT	PFTs results in summary statements for included studies
Aprian 1982 [1]	FVC	PFTs decreased in 70.3% of patients with only 30.7% showing improvement post-operatively. 22 patients (12/22 Type II SMA, 7 Type I, 3 Type III), 15 with Harrington rod instrumentation, 6 with Dwyer anterior instrumentation
Piasecki 1986 [26]	VC	VC decreased from mean 1033 mL (39% of predicted normal) pre-operatively to 860 mL (24% of predicted normal) post-operatively at mean final follow-up of 7 years
Brown 1989 [3]	FVC	The majority of patients (63%) had a decline in PFTs post-operatively, with a mean decrease of 16% of predicted FVC post-operatively, at 2 years follow-up. A small increase in PFTs (6.6% of predicted normal) was seen in 37% of patients. Tracheostomy was instituted perioperatively for 30% of patients
Merlini 1989 [23]	VC	No change in absolute VC for 5 of 6 patients with a posterior approach at last follow-up (mean, 36 months) but VC decreased by 40% for 1 patient with an anterior approach
Granata 1993 [14]	VC	VC decreased post operatively by 429 mL (23%) then returned to baseline by “6–12 months”. At final mean follow up of 5 years, the mean loss in VC was 158 mL from baseline
Robinson 1995 [28]	VC	VC improved post operatively by 21%; no change in predicted VC. Only 9/16 operated patients with complete PFTs
Chng 2003 [4]	FVC	Rate of decline of % predicted FVC by 7.7% per year pre-operatively, decreasing to 3.8% per year post operatively
Chong 2010 [5]	VC	Rate of decline of % predicted VC was 10% 1 year post operatively. During the second post-operative year, % predicted VC declined by 3%. Peak cough flow and end-tidal CO2 did not deteriorate by 1 year post-operatively
Fujak 2012 [12]	VC	VC slightly decreased post operatively, returning to baseline level after 6–12 months, then remaining stable over follow-up period. PFTs in non-operated patients also remained relatively stable over the follow-up period
Chou 2017 [6]	FVC, FEV_1_	No difference in PFTs from pre-operative to short, mid, or long term follow-up. Significant decline in PFTs between mid (5–10 years) and long term (> 10 years) follow up (FVC, FEV_1_), and between short term (< 5 years) and mid term follow-up (FVC only), post-operatively
Chua 2016 [7]	FVC	Rate of decline of % predicted FVC was 5.31% per year pre-operatively decreasing to 1.77% per year post-operatively. No effect of apical vertebra level on PFT
Lenhart 2017 [19]	FVC	Significant improvements in FVC (absolute) by 0.41L, but a gradual worsening of % predicted FVC from 48 to 35% at last follow-up. Only 6 of 16 patients (all Type II) had complete PFTs. Only 1 of 16 patients required an increase in respiratory support by last follow-up
Holt 2017 [15]	FVC, FEV_1_	Decrease in rate of decline in both FVC and FEV_1_ compared to pre-operative baseline. The rate of decline of FEV_1_ decreased from 7.2% to 2.3%. The rate of decline of FVC decreased from 6.0 to 2.9%. There was a small acute decline in PFTs in the immediate postoperative phase
Farber 2020 [10]	VC	Absolute VC declined by 0.6L (from 2.34 L) at mean 219 days at first PFT post-operatively, with a further decline of 0.48 L (from 1.86 L) at mean 399 days at second PFT post-operatively. No control group but compared to DMD and MDMD, whose PFTs mostly declined in the former and improved in the latter

*FVC* forced vital capacity, *VC* vital capacity, *FEV*_*1*_ forced expiratory volume over 1 s, *DMD* Duchenne muscular dystrophy, *MDMD* Merosin-deficient muscular dystrophy

VC measured with a pocket spirometer

Only two studies reported improvement of PFTs, with mean VC increasing by 21% post-operatively (only 9 of 16 patients had pre-operative/post-operative PFTs) in one study [28], and with absolute FVC increasing by 0.41L in a second study utilizing telescopic growing rods [19]. In this second study, however, the % predicted FVC declined from 48 to 35% at final follow-up, and only 6 of 16 children (all Type II) had both pre-operative and post-operative PFTs available.

The most common chest-related complication was atelectasis, followed by pneumonia, pneumothorax, pleural effusion, need for intubation, and pulmonary embolism. The most common encountered spine-related complications were implant failure, curve progression and pseudoarthosis. Other complications, which were reported in the included studies, were surgical site infection; wound healing problems, and death (Table 3).

**Table 3:** Post-operative complications from the included studies that reported these outcomes

Study Name	# Of patients	Spine related	Chest related	Others
Aprin 1982 [1]	22	Weakness of neck muscles: 14 Pseudoarthosis: 1 Increased kyphosis: 2 Curve progression: 1	Atelectasis: 10 Need for intubation: 4 Narrowing of chest diameter: 6	NR
Brown 1989 [3]	40	Pseudoarthosis: 3 Implant failure: 3 Lamina Fracture: 1	Plural effusion: 1 Pneumothorax: 1	SSI: 1 Spinal cord injury: 1
Merlini 1989 [23]	7	Implant failure: 2 Curve progression: 1 Pseudoarthosis: 1	Plural effusion: 1	NR
Granata 1993 [14]	18	Implant failure: 3 Progression of the curve: 1 Pseudoarthosis: 1 Symptomatic implant: 1	Plural effusion: 1 Recurrent bronchitis: 1	SSI: 1
Robinson 1995 [28]	16	Implant failure: 3	Pneumothorax: 3	Death: 1 SSI: 1
Chong 2010 [5]	11	NR	Atelectasis: 1 Pneumonia: 1	Pulmonary congestion: 1 SSI: 1 GI bleeding: 1
Fujak 2012 [12]	41	Crankshaft phenomenon: 6 Implant failure: 2	Plural effusion: 3 Atelectasis: 4 Pneumothorax: 1	Delay wound healing: 6 Spinal cord injury: 1
Chua 2016 [7]	11	NR	Pneumonia: 1.5%	Death: 2 (average age 17.5 years)
Holt 2017 [15]	16	NR	Pneumonia: 1 Re-intubation: 1	SSI: 1 Wound healing issue: 1

*SSI* surgical site infection, *GI* gastrointestinal, *NR* not reported

Due to the low level of evidence of the included studies, we are not able to make strong recommendation statements as part of this review. As such, the following GRADE C recommendations were made regarding the impact of scoliosis surgery for SMA:

1. Scoliosis surgery is most often associated with decreases in pulmonary function;
2. The impact of scoliosis surgery on pulmonary function is variable but does not reliably improve over pre-operative baseline;
3. Scoliosis surgery may result in a decreased rate of decline in pulmonary function post-operatively.

## Discussion

**Fig. 2:**
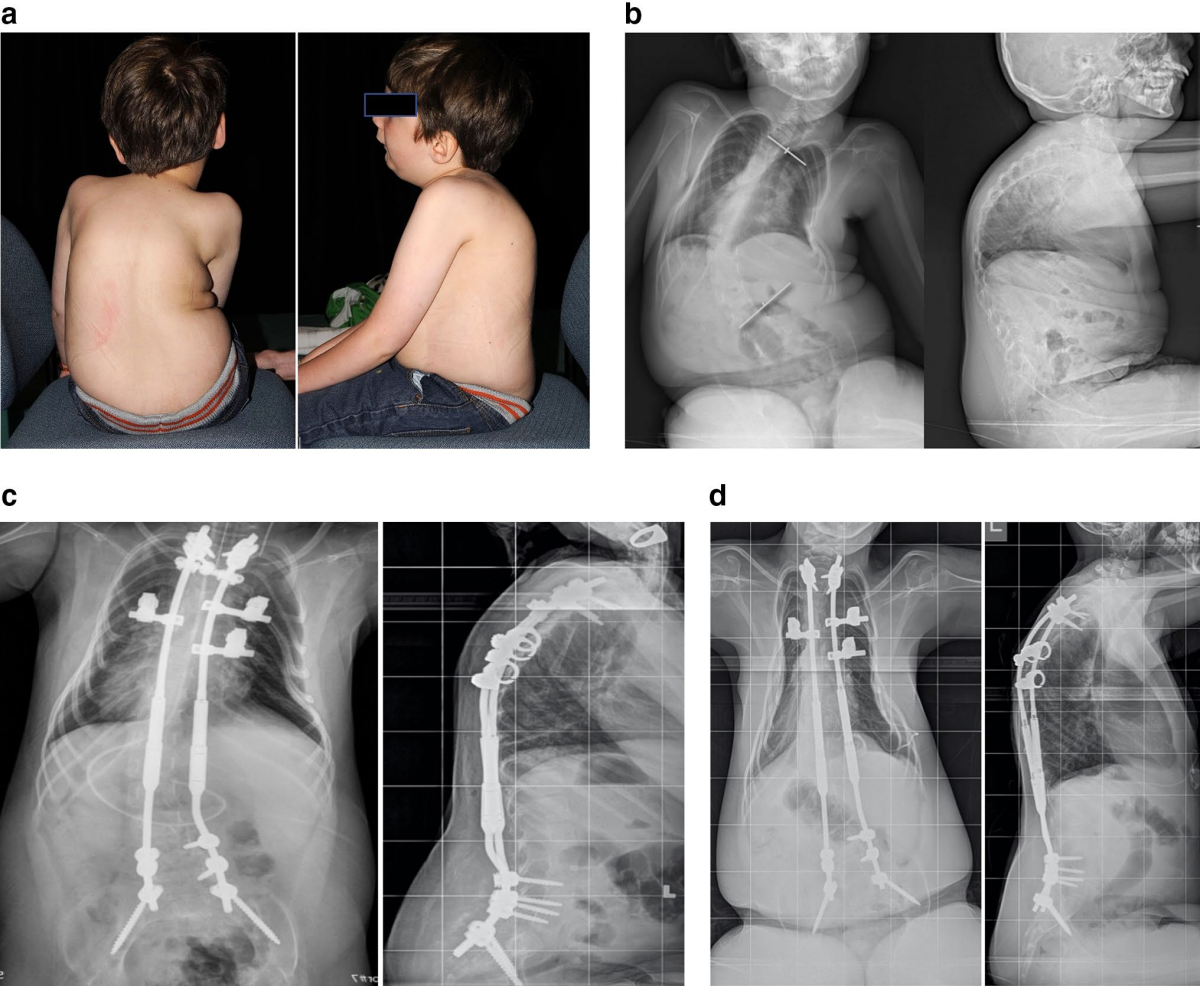
8 year-old male with Type II SMA. **a** Clinical photos. **b** Pre-operative X-rays showing progressive long C-shaped scoliosis. **c** Immediate post-operative X-rays showing hybrid construct utilizing upper thoracic pedicle screws and rib anchors, distal lumbo-pelvic pedicle screw fixation, and magnetically-controlled growing rods (MCGR). His FVC at this point was 50% predicted. **d** By three years post-operatively, his FVC has declined to less than 30% predicted. Note progressive ‘parasol’ chest deformity despite rib anchors and scoliosis correction. A recent MCGR exchange had been performed. Courtesy of Ron El-Hawary

The progressive decline in pulmonary function seen in SMA remains of major concern, the most common cause of mortality (for Types I and II) being pulmonary failure [31]. Concomitant with decreases in pulmonary function, in the pre-Nusinersen era, almost 100% of Type II and III patients will develop scoliosis by adolescence [12]. Given this temporal relationship, it is expected that a causal relationship between scoliosis progression and pulmonary function decline would be sought. As has been found in other progressive neuromuscular disorders, however, deterioration in pulmonary function may be more related to primary respiratory muscle weakness than to spinal deformity [16]. Indeed, respiratory impairment in SMA is multifactorial in origin, including: (1) intercostal muscle weakness with progressive ‘parasol’ chest deformity, (2) reduced chest wall compliance, (3) reduced lung compliance secondary to micro-atelectactic changes, (4) reduced alveolar multiplication, and (5) scoliosis causing a restrictive pattern [31]. For patients with early-onset scoliosis, impairment of alveolar development with resulting thoracic insufficiency syndrome has been suggested to be a significant contributor, despite the fact that the most common curve pattern is C-shaped and thoracolumbar with a relatively straight thoracic segment. [27, 31] Further, rather than solely from a spine-based deformity, the progressive bell-shaped ‘parasol’ chest seen in SMA is a more likely contributor to pulmonary dysfunction; with some centers employing rib-to-rib constructs to expand the thorax and increase the space available for the lung (Fig. 2). This progressive chest wall deformity due to intercostal muscle weakness has been reported to occur regardless of surgery, whether by posterior fusion or growth-friendly constructs. [2, 20, 21, 27]

In the current review, it was found that pulmonary function declined regardless of scoliosis correction for most series. Several of the included studies, however, reported a decreased rate of decline in pulmonary function from pre-operative rates which, if true, would be a desirable outcome for this progressive disorder [16, 27, 31]. Unfortunately, the methodological quality of the included studies makes it difficult to come to any firm conclusions in this regard. In the included studies, substantial sources of bias were identified, including: (1) combined analysis of SMA types with differing disease severity and progression, (2) variable follow-up periods ranging from 1.1 to 12.3 years with variable frequency and timing of post-operative PFT measurements, (3) lack of a control (non-operative) group for most studies, (4) low sample sizes with an average of 17 patients per study, (5) use of predicted PFT values rather than absolute values when reporting outcomes.

Although more than 70% of patients in the included studies were Type II, 17% were higher functioning Type III patients who would be expected to have better post-operative pulmonary function, and better tolerance for scoliosis surgery, than patients with worse disease severity. In this regard, Khirani and colleagues reported that pulmonary function in Type II patients deteriorates earlier and more rapidly that Type III patients, thus, the combined analysis of Type III with Type II (and Type I) patients in the included studies represents a substantial source of bias [18].

The timing of surgical intervention and variable periods of post-operative follow-up for the included studies is also a cause for concern. In one large case series of 126 patients (99 with SMA Type II) without surgery, the authors found that scoliosis and pulmonary function typically stabilize by adolescence, despite a more rapid deterioration in both seen at younger ages (i.e. during periods of maximal growth) [12]. With the average age at the time of surgery for included studies being approximately 12 years old, the decreased rate of decline, or stabilization of, post-operative pulmonary function reported in some of the included studies might be explained by natural history alone rather than as a direct impact of scoliosis correction surgery.

Although it is felt by some that early intervention with ‘growth friendly’ constructs will further improve pulmonary function and thus, longevity in SMA [9, 17], the literature is sparse and inconclusive at this point. Only two of the included studies analyzed patients that could truly be considered “early-onset” from a neuromuscular scoliosis point-of-view (i.e. less than 8 years old). Lenhart and colleagues reported a series of 16 patients with Type I and II SMA treated with traditional dual growing rods, at an average age of 5.8 years [19]. Although they found an increase in absolute FVC over 4.7 years follow-up post-operatively, only 6 of 16 patients had complete PFTs for review. In addition, they found a gradual deterioration in percent-predicted FVC values despite the increase in absolute values, highlighting the problems with comparing these two types of measurement as suggested above. Fujak and colleagues found no significant difference between operated (including both posterior fusions and growing “telescopic” rods) and non-operated patients, but those treated with telescopic rods, paradoxically, had a larger pulmonary decline over time. Given that these children required scoliosis surgery at an earlier age than those with posterior fusions (mean, 6.7 years vs 12.3 years), this decrease could be more related to disease severity than to the surgical approach. That said, the authors did describe a higher prevalence of crankshaft and loss of scoliosis correction in the telescopic rod group due to inadvertent dorsal fusion and high implant-related complications. Clearly, higher quality, controlled studies are needed to answer the question as to whether the use of growth friendly constructs for early onset scoliosis in SMA is warranted, taking into account the associated risk–benefit balance.

As for risks, pulmonary complications including pneumonia, pneumothorax, pleural effusion, and need for re-intubation post-operatively, were relatively common in the included studies, as were spine-related complications (Table 3), consistent with larger samples [2]. Accordingly, the indications and goals of scoliosis correction need to be clarified, to ensure that the benefits outweigh the risks in this medically fragile population. For other neuromuscular diagnoses [including Duchenne Muscular Dystrophy (DMD)], the goals of surgery have shifted to those relating to the quality of life, including issues such as comfortable sitting, positioning, and appearance, as scoliosis correction has not reliably led to improved respiratory outcomes [8, 24]. Given the low level of evidence provided by the included studies in this review, there is currently insufficient evidence to support the application of scoliosis surgery to improve pulmonary function in SMA.

Newer therapies such as Nusinersen (marketed as Spinraza®, Biogen, Cambridge, MA, USA) and gene therapies such as Zolgensma® (Novartis, Bannockburn, IL, USA), represent a changing paradigm in SMA treatment and disease trajectory that need to be considered. These therapies have resulted in marked improvements in gross motor function and have decreased the need for mechanical ventilation in these patients [8]. None of the studies in the current review assessed the impact of Nusinersen and thus, its influence on the development and progression of scoliosis in SMA is not yet known.

There were limitations to this review, most notably the low level of evidence available for synthesis—the majority being case series with small sample sizes without a comparator group. As a result, the GRADE recommendations could only be given at a C level. In addition, the heterogeneity in study design and reported pulmonary outcome measures precluded our ability to perform a meta-analysis.

In conclusion, the literature supporting the application of scoliosis surgery to improve pulmonary function in SMA at present is poor. Well designed, preferably prospective, studies are urgently needed to determine the role of scoliosis correction in this regard, especially with the advent of new disease-modifying agents which may have an even bigger impact. In the meantime, practitioners should temper their expectations with respect to the reliability of pulmonary function improvement post scoliosis surgery, and counsel their patients accordingly regarding the current state of the literature.

## Data Availability

Available upon request.

## Code availability

Available upon request.
